# On Aconite in Dysentery

**Published:** 1850-03

**Authors:** 


*On Aconite in Dysentery.* By M. Marbot.—M. Marbot, surgeon-major of the Crocodile man-of-war, found himself in the midst of an epidemic of dysentery, a few days after the vessel had left Zanzibar, nearly every one on board being sooner or later attacked during its two months' continuance. At first a condition of *embarras gastrique* prevailing, the cases were successfully treated by ipecacuanha and sulphate of soda; but the vessel returning into the warmer latitudes, the affection assumed an inflammatory type. There was intense fever, hard, contracted, rapid pulse, violent headache, a dry and bitter mouth, though the tongue was flattened and not much-loaded; tenderness of the abdomen, colic, distressing pulsation in the right hypochondrium, and tenesmus were present. The emetico-purgative plan now entirely failed, and M. Marbot feared the necessity of bloodletting, of the general inutility of which, in hot climates, he had had frequent opportunities of judging. He however bethought himself of *aconite*, from which he had derived great benefit in acute rheumatism. Its effects quite surpassed his expectations, for the inflammatory excitement subsided in less than a day, and the blood disappeared from the stools in a few hours. From this time he gave the remedy even from the commencement of the disease, and he always found it remove the hemorrhage and abate the fever, the pain in the belly, too, becoming relieved, and the stools passing easier, even a few hours after the first dose. But the aconite exerts no other effects upon the stools than removing the blood from them, their mucous, glairy, &c. characters continuing as before, and even their number not undergoing a diminution proportionate to the improvement of other symptoms. The aconite, then, would seem to exert a very feeble action on the intestinal contractions, but promptly subdues the febrile reaction and the excitement produced in the various organs. The dose required is not large, being but from five to ten centigrammes of the extract in the twenty-four hours, diluted in water, and given in fractional portions every two or three hours.

The aconite does not *cure* the dysentery, but so modifies its nature, as to render it amenable to treatment that before proved useless. Thus, as soon as the reaction is reduced, M. Marbot has at once recourse to ipecacuanha, allowing a day to intervene between each dose. After the stools become somewhat reduced in number, we may follow up the advantage, by the use of the starch and opium clysters. Mercury should be substituted for ipecacuanha, when hepatitis or a disturbance of the secretions of the liver or pancreas is present, and the stools are found green, opaque, or foamy, i. e. muco-purulent. Opiates are useful only after the above-named remedies have produced their effects, and are injurious as long as any inflammatory action is present. Quinine is useful in hot climates, when the disease is masking a remittent.

Upon these principles M. Marbot treated his 300 cases, some of which were of the severest character, and others attended by relapses, without losing a patient. A trial made in Paris of this remedy leads to the belief that it may be advantageously used to render the evacuations in dysentery and diarrhœa less irritating, and for the relief of the febrile reaction set up at the end of phlegmasiæ. The extracts prepared from the plant growing near Paris, and from that growing on the Alps, differ so much in activity, that it is recommended whenever the extract seems not to avail, to substitute the tincture, as a more certain preparation.—*Brit. and For. Med. Chir. Rev. from Bulletin de Therapeutique.*

